# Interocular contrast difference drives illusory 3D percept

**DOI:** 10.1038/s41598-017-06151-w

**Published:** 2017-07-17

**Authors:** Alexandre Reynaud, Robert F. Hess

**Affiliations:** McGill Vision Research, Dept. Ophthalmology, McGill University, Montreal, QC Canada

## Abstract

Any processing delay between the two eyes can result in illusory 3D percepts for moving objects because of either changes in the pure disparities over time for disparity sensors or by changes to sensors that encode motion/disparity conjointly. This is demonstrated by viewing a fronto-parallel pendulum through a neutral density (ND) filter placed over one eye, resulting in the illusory 3D percept of the pendulum following an elliptical orbit in depth, the so-called Pulfrich phenomenon. Here we use a paradigm where a cylinder rotating in depth, defined by moving Gabor patches is presented at different interocular phases, generating strong to ambiguous depth percepts. This paradigm allows one to manipulate independently the contrast and the luminance of the patches to determine their influence on perceived motion-in-depth. Thus we show psychophysically that an interocular contrast difference can itself result in a similar illusory 3D percept of motion-in-depth. We argue that contrast, like luminance (ND filter) can modify the dynamics of visual neurons resulting in an interocular processing delay or an interocular velocity difference.

## Introduction

Any processing delay between the two eyes can result in illusory 3D percepts for moving objects because of either changes in the pure disparities over time for disparity sensors^1^ or by changes to sensors that encode motion/disparity conjointly^2^. This is demonstrated by viewing a fronto-parallel pendulum through a neutral density filter placed over one eye, resulting in the illusory 3D percept of the pendulum following an elliptical orbit in depth, the so-called Pulfrich phenomenon^3^.

The monocular delay induced by luminance^4^ thought to be responsible for the Pulfrich phenomenon has been observed in several species at the level of the retina^5^, and at the cortical level for both single cell^6^ and population scales^7, 8^. In human and non-human primates, binocular neurons in MT, encoding motion-in-depth are sensitive to disparity, interocular velocity differences and delays^9–15^, similar to that generated by a monocular luminance decrement, suggesting that the delay induced by a luminance decrement is responsible for the perception of illusory motion-in-depth in the Pulfrich phenomenon^16, 17^.

However, a luminance decrement is not the only way to increase response latency. From the retina^18^, LGN^19^, V1^20^ to MT^21^, and from the single cells to populations^22^ and behavior^23^, it has been observed that a decrement of the stimulus contrast, will also result in an increment in the response latency. Thus, if the binocular cells, integrating the signal coming from the two eyes in the cortex are responsible for the Pulfrich phenomenon because of the delay generated by luminance in one of the monocular streams^16, 17^, it should be possible that a similar delay, generated by a contrast decrement would generate a similar percept. The binocular fusion of stimuli with different contrasts in the two eyes is possible, with a higher weight attributed to the eye with the highest contrast stimulus^24–27^. This change in weighting could be accompanied by a change in the processing time of each eye’s input during the binocular combination.

**Figure 1:**
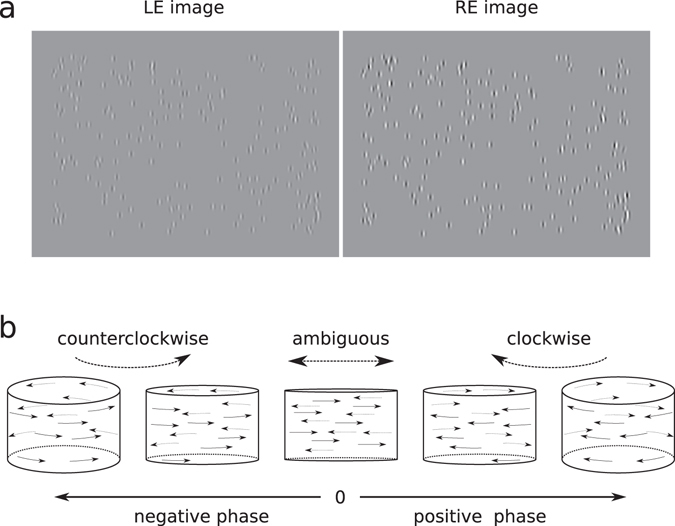
Stimuli. (**a**) The stimulus consists of 200 Gabor patches oscillating horizontally and presented dichoptically with different contrasts to each eye (here left eye 40%, right eye 80%). (**b**) The phase difference in the oscillation of the Gabor patches between the two eyes generates a percept of a motion-defined cylinder rotating in depth. If the phase difference is negative, the cylinder is seen rotating anticlockwise, with the front plane of the cylinder going to the right. If the phase difference is zero, the percept is ambiguous with Gabor patches moving to the left and to the right in the same plane. If it is positive the cylinder is seen rotating clockwise. Videos of a simplified version of the stimulus are available as supplementary material.

So far, studies that have focused on a depth perception induced by a monocular contrast change have used random dot patterns in which the background or inducing luminance was manipulated resulting in a combined change in luminance and contrast^28, 29^. In this study, we use a paradigm where a cylinder rotating in depth, defined by moving Gabor patches is presented at different interocular phases, generating strong to ambiguous depth percepts (Fig. 1, supplementary movies)^12, 30^. When viewed monocularly or with a zero interocular phase, individuals will likely experience a motion-in-depth percept known as the kinetic depth effect^31, 32^. However, the low-level interocular phase manipulation creates an actual disparity. This paradigm involves structure-from-motion and depth perception mechanisms^12^ and allows one to manipulate independently the contrast and the luminance of the patches. This allows for the precise measurement of the interocular delay in terms of the phase difference between the two eyes’ images at the point of subjective equality (PSE) where the cylinder’s clockwise and anticlockwise rotation is ambiguous. The subjects’ task was to report whether they saw the cylinder rotating clockwise or anticlockwise. The interocular phase difference was manipulated for different interocular luminance or contrast conditions.

## Results

**Figure 2:**
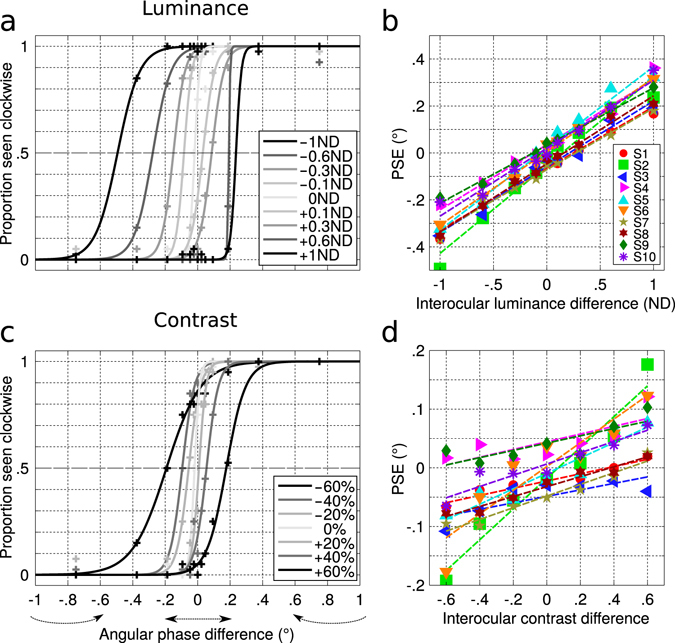
Results. (**a**) Psychometric functions of the perceived direction of rotation for one subject (S2) as a function of the interocular phase difference for different luminance conditions. Negative ND value: indicates filter is placed in front of the left eye, positive: filter placed in front of the right eye. Datapoints are fitted by a logistic function. The midpoint of the logistic function at 0.5 performance defines the point of subjective equality (PSE). (**b**) PSE as a function of the interocular luminance difference for the individual 10 subjects. Dashed lines represent linear regression. (**c**) Psychometric functions of the perceived direction of rotation for the same subject as a function of the interocular phase difference for different contrast conditions. Negative contrast value: contrast decreased in the left eye image, positive: contrast decreased in the right eye image. (**d**) PSE as a function of the interocular contrast difference for the individual 10 subjects.

In Fig. 2a, the psychometric functions are displayed for one subject who reported a clockwise perception as a function of the interocular delay for different luminance conditions. One can see that the psychometric function is shifted to the left when the luminance is decreased over the left eye which means that the cylinder is seen more often rotating clockwise; and that it is shifted to the right when the luminance is decreased over the right eye, meaning that the rotation is seen more anticlockwise. The estimated PSE for changes in the interocular phase is then plotted as a function of the interocular luminance difference in Fig. 2b for all subjects. In the viewing condition with no luminance imbalance (0ND), most subjects’ PSE are offset from 0°, characterizing a “spontaneous Pulfrich phenomenon” in the normal population not previously observed^33^. When a ND filter is placed in front of one eye, the PSE on average increases from −0.32° at −1ND to 0.26° at + 1ND interocular luminance difference, which corresponds to an average interocular delay of 16 ms. The data are fitted by linear regressions with slopes that are significantly positive for all subjects (mean slope 0.29 ± 0.04, right-tailed Wilcoxon signed rank test, *p* < 0.001). These results confirm what has been reported by Carkeet *et al*.^34^, namely that the interocular temporal asynchrony is dependent on the interocular luminance difference and is symmetrical whether the left or right eye luminance is decreased. Therefore, the amplitude of the luminance-induced Pulfrich phenomenon depends on the interocular luminance difference.

If the depth percept arises from a luminance-induced neural delay, then this phenomenon should be reproducible with a monocular contrast decrement which will also delay the neural processing of one eye. In Fig. 2c, we display psychometric functions similar to that already described in Fig. 2a, but for interocular contrast differences. Here again, we can see that the cylinder is seen more often rotating clockwise when the contrast is decreased in the left eye image; and that it is seen more often rotating anticlockwise when the contrast is decreased in the right eye image.

In Fig. 2d, we plot the estimated PSE for the interocular phase difference as a function of the interocular contrast difference for all subjects, similar to that for luminance in Fig. 2b. On average, it increases from −0.08° at −60% to 0.07° at + 60% interocular contrast difference, which corresponds to an interocular delay of 4 ms. The datapoints are fitted by linear regressions with significantly positive slopes for all subjects (mean slope 0.11 ± 0.07, right-tailed Wilcoxon signed rank test, *p* < 0.001). These results are novel and show that the interocular temporal asynchrony is dependent on the interocular contrast difference, in a symmetrical way for the left or right eye decrements in contrast. This effect is much smaller than the one observed for luminance reductions but it shows that an interocular contrast difference can generate a Pulfrich phenomenon for a moving stimulus.

**Figure 3:**
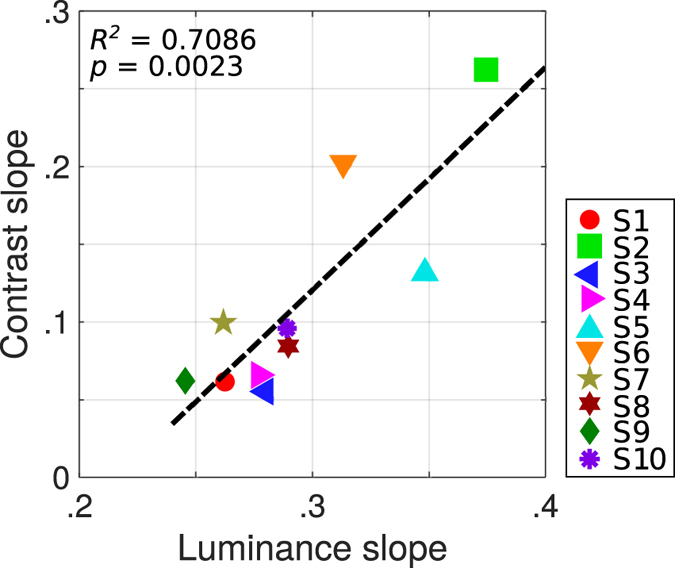
Correlation between luminance- and contrast-induced phenomenons. Regression slopes of the angular PSE for the interocular contrast difference as a function of the slopes of the angular PSE for the interocular luminance difference for the 10 subjects. The dashed line represents the least square regression: *R* ^*2*^ = 0.7086, *p* = 0.0023.

To investigate if the effects induced by luminance and contrast share a common mechanism, we report the correlation between the regression slopes of the angular PSE as a function of interocular contrast and luminance difference in Fig. 3 for the 10 subjects. This correlation is strong with a coefficient of determination *R* ^*2*^ = 0.7086 and *p* = 0.0023 indicating that these two effects likely share a common mechanisms.

## Discussion

Spatio-temporal phase shifts can generate a depth perception for moving stimuli^35–37^. This provides the basis for our cylinder stimulus seen rotating in depth when the interocular phase difference is changed. With this protocol, we show that a contrast imbalance between the two eyes can produce an illusory percept of motion-in-depth.

When seeing ambiguous planar motion, individuals will likely experience a motion-in-depth percept due to a bias which could be driven by the kinetic depth effect^31, 32^. The contrast-dependent phenomenon reported here is very sensitive and can interact with subjects’ innate bias. In Fig. 2d, for all subjects, the PSE shows a clear increasing trend as a function of the interocular contrast difference. The PSE at 0 doesn’t deviates from the trend and, in some cases, this innate bias cannot even be reversed by a small contrast or luminance imbalance (see also Fig. 2b). For some subjects (e.g. S3, S4, S9), it even remains consistently always positive or negative, because the innate bias is stronger than the induced stimulus contrast imbalance. Furthermore, if the phase is kept at 0° (ambiguous case) and only the contrast is changed, some subjects will perceive the stimulus always rotating in the same direction regardless of the contrast imbalance of the stimulus because of the more dominant influence of their innate bias (see Supplementary Figure 1). This could provide an explanation for why a contrast-induced interocular delay has never been observed previously. Due to its consistency over the contrast and luminance range, we therefore suspect this bias to be due to a low-level innate contrast imbalance between the two eyes, revealing a “spontaneous Pulfrich phenomenon”^33^ rather than being perceptual (the kinetic depth effect). However we cannot totally rule out this possibility.

We observe a larger variability in the slope as a function of the interocular difference in the contrast condition than in the luminance condition. It could be due to the innate variability in the contrast sensitivity of the participants (see Supplementary Figure 2) which falls within the normal range^38^.

We estimated that a fourfold contrast reduction in the high contrast range induced a delay of approximately 4 ms. This value falls in the same range as what has been observed in monkey primary visual cortex at both the single cell level^20, 39, 40^ and the population level^41, 42^. The same kind of delay as a function of contrast has been reported for perceptual judgment^43^ and in the microsaccade onset time in humans^44^. Thus, we hypothesized that a contrast-generated delay in neural processing is responsible for the illusory depth percept. Also the correlation we observed between the slopes of the contrast- and luminance-based effect could indicate that they are both mediated by a common contrast-gain control mechanism and hence, that the Pulfrich phenomenon might be partially mediated by a contrast-gain control mechanism. This result could then open new ways of interpretation of this phenomenon. However, an alternate explanation could be advanced that the motion-in-depth could actually be computed via an interocular velocity difference^45–47^. It is known that a reduction of contrast results in the perception of objects moving at a slower speed whether they are moving in depth^48^ or within a plane^23, 49^. Thus the reduced contrast Gabor patches seen by one eye could be monocularly perceived as moving more slowly than the high contrast ones seen by the other eye. However, Campbell and Maffei^50^ reported that a grating is only perceived to rotate more slowly when the contrast is below 0.05, much lower than the contrast for which the effect is observed here. An interocular speed difference could generate a motion-in-depth percept once the two monocular trajectories are combined. In fact, if we assume that the luminance reduction by the use of ND filters does not simply generate a delay but instead result in a low-pass filtering of the input^51^, then, an induced slowing down of one eye image could also induce an interocular velocity difference which could provide an alternate explanation for the classic Pulfrich phenomenon. Whatever the explanation, we have shown for the first time that contrast as well as luminance imbalances can drive illusory percepts of depth.

## Methods

### Stimuli

The stimulus was a structure-from-motion defined rotating cylinder of 18° width and 12° height, consisting of 200 Gabor patches oscillating horizontally at a sinusoidal angular speed of 18°/s for a duration of 800 ms (Fig. 1a, supplementary movies). The stimulus was presented dichoptically. The interocular phase of the oscillation was consistent between all Gabor patches trajectories and was varied to generate strong to ambiguous percepts of a cylinder rotating in depth (Fig. 1b)^12, 30^. Each Gabor patch was 0.15° sigma, 2.85 c/d spatial frequency with a random phase. Stimuli and experimental procedures were programmed with Matlab R2015a (© the MathWorks) using the Psychophysics toolbox^52–54^. Experiments were run on an Apple MacPro computer with a Nvidia GeForce 8800 GT graphics card, running on Linux Mint operating system. The stimuli were presented on a wide 23” 3D-Ready LED monitor ViewSonic V3D231, gamma corrected with a mean luminance of 100 cd.m^−2^ at a resolution of 1920 × 1080 px and a refresh rate of 60 Hz in interleaved line stereo mode. The subject viewed the stimuli at a viewing distance of 90 cm, in a dim-lit room, with passive polarized 3D glasses which had the effect of reducing the luminance to about 40%, measured with a photometer and a crosstalk of 1%^55^.

### Procedures

10 subjects (4 males, mean age 26.8 ± 6.5 yo, one author: S1, their monocular contrast sensitivity are reported in Supplementary Figure 2) with normal stereo vision (all thresholds below 100 seconds, randot test) participated in this experiment. Their task was to report whether they saw the cylinder rotating clockwise or anticlockwise as a function of the interocular phase in a block design paradigm. In one block, one contrast or one luminance was tested with 10 repetitions. Each block was repeated 2 times in a counterbalanced way. Figure 2a depicts the psychometric function of one subject reporting a clockwise perception as a function of the interocular phase difference for different luminance or contrast conditions. Luminance modulations were generated by placing a ND filter in front of one eye (negative values indicate the filter was in front of the left eye, positive values in front of the right eye). Contrast (expressed as Michelson’s) was modulated on-screen (negative values, contrast decreased in the left eye image, positive values contrast decreased in the right eye image). This research has been approved by the Ethics Review Board of the McGill University Health Center and was performed in accordance with the ethical standards laid down in the Code of Ethics of the World Medical Association (Declaration of Helsinki). Informed consent was obtained from all subjects.

### Data analysis

The data was analyzed with Matlab R2014a (© the MathWorks). The psychometric functions were fitted with a logistic function forced between 0 and 1. The estimated midpoint of the logistic function defines the point of subjective equality (PSE), the point at which the perception is 0.5 clockwise and 0.5 anticlockwise. The estimated point of subjective equality is then reported as a function of the interocular luminance difference and fitted by a linear regression in a fashion similar to Carkeet *et al*.^34^.

## Electronic supplementary material

supplementary material

Video 1

Video 2
