# Supplementary material for: Interocular contrast difference drives illusory 3D percept
Source: Sci Rep. 2017 Jul 17;7:5587. doi: 10.1038/s41598-017-06151-w (PMC5514099; doi:10.1038/s41598-017-06151-w)
Supplement: Supplementary file 1 — supplementary material [file 41598_2017_6151_MOESM1_ESM.pdf]

Interocular contrast difference drives illusory 3D percept

Supplementary Information

Alexandre Reynaud and Robert F Hess

McGill Vision Research, Dept. Ophthalmology, McGill University, Montreal, QC, Canada

Supplementary Figure 1:

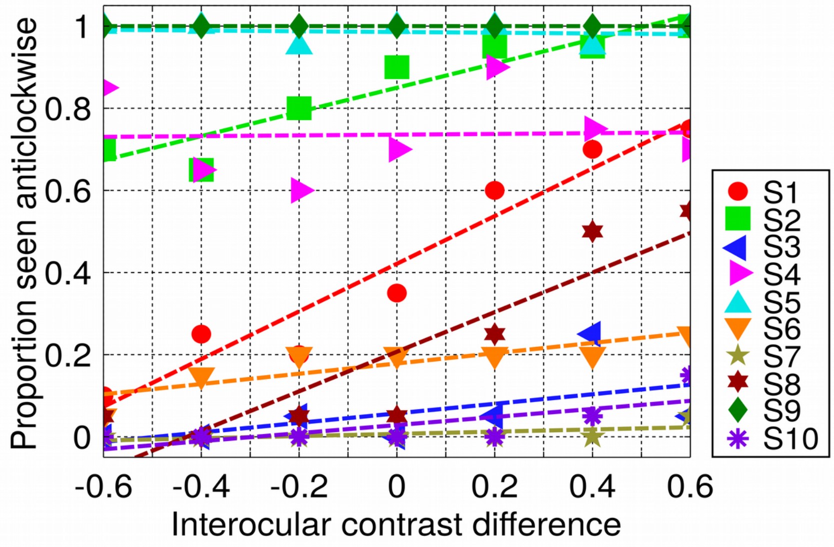

Supplementary Figure 1 shows the results of a control experiment where subjects reported the perceived direction of rotation as a function of the interocular contrast when there is no interocular phase difference (dashed lines represent linear regression). In that case, most subjects see the cylinder almost always rotating in the same direction, either anticlockwise or clockwise with proportions seen clockwise close to 0 or 1 respectively. Only 4 subjects (S1, S2, S6 and S8) show a significant trend in the perceived direction of rotation as a function of the interocular contrast ($p<0.05$). The curve crosses the 0.5 proportion midline for only 2 of them (S1 and S8). Hence such a protocol can hardly reveal the contrast-induced depth illusion reported here and we think this is the main reason why this effect has never been observed.

Supplementary Figure 2:

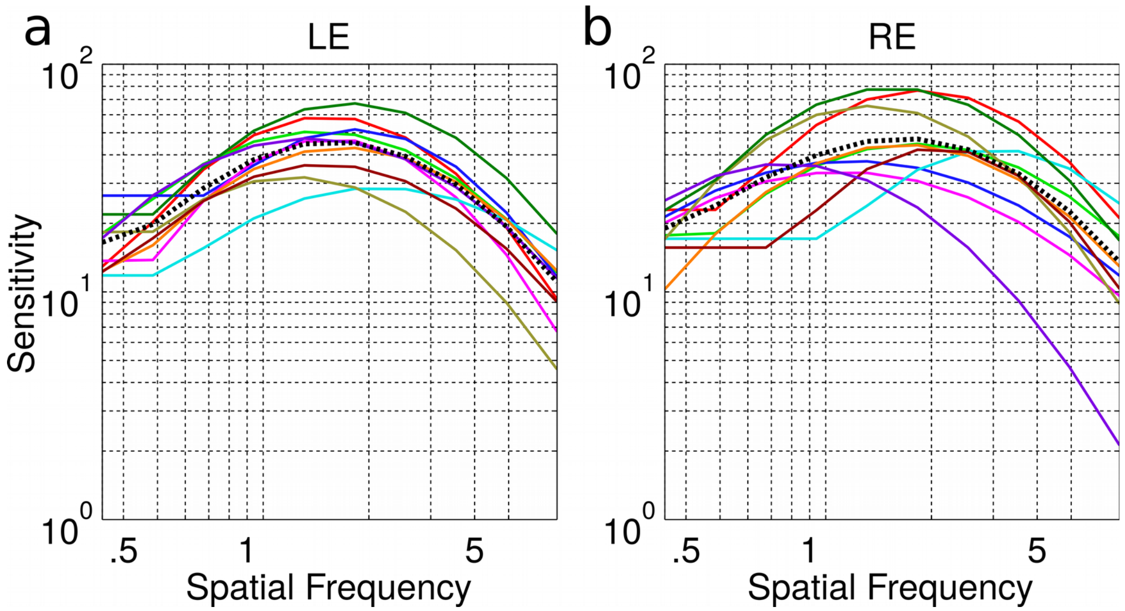

Supplementary Figure 2 illustrates the monocular contrast sensitivity as a function of the spatial frequency for the left (a) and right eye (b) of all participants (same color-code as Supplementary Figure 1) measured with the quick Contrast Sensitivity Function (qCSF, Lesmes et al., 2010) with the same apparatus and procedure as used in Reynaud and Hess, 2016).

Supplementary Videos:

Supplementary Videos 1 and 2 show a not-to-scale version of the stimulus presented with an interocular phase difference making the stimulus appear to rotate counterclockwise with the same contrast (80% contrast in each eye: Supplementary Video 1) or different contrasts (40% and 80%: Supplementary Video 2) in each eye images. These videos can be free-fused, viewed with a stereoscope or displayed on a 3D screen or head-mounted display.

References:

- Lesmes, L. A.; Lu, Z.-L.; Baek, J. & Albright, T. D. 2010. Bayesian adaptive estimation of the contrast sensitivity function: the quick CSF method. *J Vis*, **10**, 17.1-1721
- Reynaud, A. & Hess, R. F. 2016. Is suppression just normal dichoptic masking? Suprathreshold considerations. *Invest Ophthalmol Vis Sci*, **57**, 5107-5115
